# EHMTI-0245. Some clinical characteristics and prophylactic treatment in patients with moh in an out-patient clinical sample

**DOI:** 10.1186/1129-2377-15-S1-G17

**Published:** 2014-09-18

**Authors:** M Jovanovic, N Buder

**Affiliations:** 1 Epilepsy and Clinical Neurophysiolofy, Institute of Mental Health, Belgrade, Serbia

## Introduction

Medication Overuse Headache (MOH) is a well-known condition and diagnostic entity, recognized as a relevant medical and socio-economic problem everywhere. Whether initial primary headache type, its frequency, co-morbid conditions or else, it is not truly clear. The therapeutic management of MOH is often difficult and still not properly and step-by-step guided.

## Aims

We wanted to point out and compare some clinical features of patients with MOH, migraine and tension type headache, as well as their response to prophylactic treatment.

## Methods

We considered a group of 100 out-patients, adults and both sexes, according to headache diagnosis (ICHD-III) - migraine (M), tension type headache (TTH), co-existence of M and TTH (M+TTH) and MOH, previous headache in MOH group and attack frequency. Therapeutic response to applied various prophylactic single or combined medications (from the Guideline of EFNS) was estimated as an initial response and/or in a followed-up period of 6 months.

## Results

There were 74 females and 26 males, 38 with M, 23 TTH, 20 M+TTH and 19 with MOH (19%) - 7 previously had M, 5 TTH and 7 M+TTH. 40 patients had less and 60 more than 15 headache days per month. Of 94 patients treated by prophylactic medication 74 (78,7%) responded and 20 (21,3%) did not - 3 had M, 8 TTH, 3 M+TTH and 6 MOH.

## Conclusions

Focused MOH group and specially those poor responders to prophylactic treatment did not show any significant differences compared to other, mostly chronic, primary headache. Although difficult to manage, MOH is a treatable condition.

No conflict of interest.

